# The influence of child and adolescent maltreatment on personality disorders and criminal activity in adult women

**DOI:** 10.1192/j.eurpsy.2022.1549

**Published:** 2022-09-01

**Authors:** M. Kachaeva, N. Kharitonova, N. Semenova, O. Rusakovskaya, V. Vasyanina, O. Shishkina, N. Skibina, L. Nazarova

**Affiliations:** 1 V. Serbsky National Medical Research Centre for Psychiatry and Narcology, Forensic Psychiatry, Moscow, Russian Federation; 2 Moscow Research Institute of Psychiatry, Clinical Psychology Counseling, Moscow, Russian Federation; 3 V. Serbsky National Medical Research Centre for Psychiatry and Narcology, Forensic Psychiatry In Civil Process, Moscow, Russian Federation; 4 I.M.Sechenov First Moscow State Medical University, Forensic Psychiatry, Moscow, Russian Federation

**Keywords:** crime, women, victims of maltreatment, personality disorder

## Abstract

**Introduction:**

Since the rates of female criminality are rising it would appear important to conduct the study of the relationship between criminal behaviour and psychiatric diagnoses in female offenders.

**Objectives:**

The main purpose of this investigation is to find out origins of crimes in women and to reveal the influence of child and adolescent maltreatment on personality disorders in adult women.

**Methods:**

Clinical psychopathological, psychological, statistical.

**Results:**

A cohort of 13 females with diagnosis of personality disorders was examined. All of them had committed crimes of violence. In the majority of the sample women had a previous history of psychiatric admissions (child psychiatric hospitals, adolescent units). The retrospective review revealed that the majority of women in their childhood were exposed to emotional, physical and sexual abuse in their families. Our results point that maltreatment may distort personality formation and social adjustment and contribute to behaviour problems, negative relation to socialization and criminal behaviour in adulthood.

**Conclusions:**

The study revealed that psychiatric disorders in childhood and adolescence are predictive of adult criminality in females and also revealed the risk of girls who are victims of maltreatment to become a perpetrator in adulthood. This findings may be used as prognostic indicators of development of aggression in female forensic patients.

**Disclosure:**

No significant relationships.

